# Chikungunya – an emerging infection in Bangladesh: a case series

**DOI:** 10.1186/1752-1947-8-67

**Published:** 2014-02-23

**Authors:** Rashedul Hassan, Md Mujibur Rahman, Md Moniruzzaman, Abdur Rahim, Satyajit Barua, Rajib Biswas, Pijous Biswas, Syed Ghulam Mogni Mowla, MA Jalil Chowdhury

**Affiliations:** 1 Department of Medicine, Shaheed Suhrawardy Medical College Hospital, Dhaka, Bangladesh; 2 Department of Medicine, Bangabondhu Sheikh Mujib Medical University, Dhaka, Bangladesh

**Keywords:** Chikungunya fever, Arthropod borne, Arthralgia, IgM antibody

## Abstract

**Introduction:**

Chikungunya is an arthropod-borne virus endemic to Africa, Southeast Asia and India that causes acute febrile polyarthralgia and arthritis. In this short case series, we discuss six Bangladeshi patients with chikungunya fever. Though Bangladesh is in endemic zone, it is not common here, hence it demands attention for proper diagnosis and management.

**Case presentation:**

The six cases of chikungunya we report occurred in native Bangladeshi women with ages ranging from 20 to 50 years and all having a middle class family background. Three women had severe incapacitating arthralgia as well as a maculo-papular rash and a high fever. The other three had a high grade fever and arthralgia only, but no rash. They were tested for chikungunya immunoglobulin M antibody and found to be positive in all cases. They were treated symptomatically with non-steroidal anti-inflammatory drugs and found responsive in most cases.

**Conclusion:**

From this case series, it is evident that chikungunya is not that uncommon in Bangladesh. But the concomitant presence of other arthropod-borne infections with similar courses of illness makes most physicians less aware of this infection. An awareness and clinical knowledge are necessary to diagnose chikungunya infection properly.

## Introduction

Chikungunya fever is a mosquito-borne illness of humans caused by the chikungunya virus, a type of alphavirus. *Aedes aegypti* and *Aedes albopictus* mosquitoes are the main vectors of chikungunya in Asia and the Indian Ocean islands. The virus was first reported in 1952 in Tanzania. Since then it has been attributed to many outbreaks in a number of countries. The virus is geographically distributed in Africa, Southeast Asia and India. Sporadic cases are regularly reported from different countries in the affected regions [1]. During December 2008, an investigation team from the Institute of Epidemiology, Disease Control and Research (IEDCR) and International Centre for Diarrhoeal Disease Research, Bangladesh (ICDDR,B) investigated the first outbreak of chikungunya fever in the Rajshahi and Chapianawabganj districts of Bangladesh [2], which was in fact the third outbreak [3] in the whole of Bangladesh. We identified six cases of chikungunya fever in the last year.

## Case presentation

Six cases of chikungunya fever were diagnosed last year in a tertiary teaching hospital in Dhaka city (Table 1). Half of our patients (three out of six) came from different parts of Dhaka city and the rest from different rural areas of Bangladesh. All our patients were native Bangladeshi women and five were housewives. Their age ranged from 20 to 50 years. All of them presented with severe incapacitating arthralgia or arthritis of variable duration (two to three weeks) with morning stiffness. They gave a history of three to five days’ high grade fever with or without a maculo-papular rash at the onset of illness. No one came to us in an acute stage and initially all were treated symptomatically for a common viral fever by local physicians. All patients were thoroughly examined and displayed multiple peripheral joint arthritis. All had high erythrocyte sedimentation rates (ESRs) (>30mm in the first hour). Anti-chikungunya immunoglobulin (Ig) M antibody (Ab) was positive and serum rheumatoid factor (RF) and anti-citrullinated protein (CCP) antibody were negative in all cases. They were treated with nonsteroidal anti-inflammatory drugs (NSAIDs) and responded well. Two of our patients had residual joint pain for two to three months after recovering from the initial infection.

**Table 1:** Summary of findings

**Findings**	**Case-1**	**Case-2**	**Case-3**	**Case-4**	**Case-5**	**Case-6**
Age (year)	45	45	20	35	50	42
Sex	F	F	F	F	F	F
Occupation	Housewife	Housewife	Student	Housewife	Sousewife	Housewife
Duration of fever						
at onset (days)	5	3	3	5	4	6
Maculo-papular						
Rash at onset	Yes	No	Yes	No	Yes	Yes
Arthralgia and/or Arthritis	Yes	Yes	Yes	Yes	Yes	Yes
ESR (mm in first hour)	65	90	50	60	30	45
RF (IU/ml)	<8.1	-	<8	<10	<8	<10
Anti CCP (IU/ml)	<10	-	<10	<11	<5	<6
Anti-chikungunya						
IgM Ab	+ve	+ve	+ve	+ve	+ve	+ve
Residual joint pain						
persisting two to three months	Yes	No	No	No	No	Yes

ESR, erythrocyte sedimentation rate; RF, Rheumatoid factor; Anti CCP, Anti-cyclic citrullinated peptide; IgM, Immunoglobulin M; Ab, Antibody.

## Discussion

The name chikungunya is derived from a local language of Tanzania meaning "that which bends up" or "stooped walk" because of the incapacitating arthralgia caused by the disease. Multiple outbreaks beyond West Africa have been described. Since 2004 chikungunya has spread widely, causing massive outbreaks with explosive onset in the Indian Ocean region, India and other parts of Asia [4,5]. The first outbreak in Bangladesh was observed in December 2008 when 32 cases were identified [3]. Since then sporadic cases were reported from different parts of Bangladesh. Our case series is an example of sporadic chikungunya infection.

The most significant manifestation of chikungunya fever is the severe joint pain occurring in virtually every clinical case [6]. In our cases, we observed the same manifestation. The arthralgia is most commonly symmetrical and peripheral, being noted in the small joints of the hands and other large joints. The joints exhibit extreme tenderness and swelling with patients frequently reporting incapacitating pain that lasts for weeks to months. Most infections completely resolve within weeks or months but there have been documented cases of chikungunya fever-induced arthralgia persisting for several years [7]. In our case series only two patients had residual joint pain for two to three months after recovering from the initial infection.

The symptoms of infection are quite similar to those caused by many other infectious agents in the endemic areas. One particular difficulty in identifying infection is its overlapping distribution with dengue virus. It has been postulated that many cases of dengue virus infection are misdiagnosed and in practice the incidence of chikungunya infection is much higher than reported [8]. Diagnosis of chikungunya is based on two cardinal signs in the acute phase; fever and arthralgia has a specificity of 99.6% and positive predictive value of 84.6% [9]. However, as the clinical manifestations of chikungunya fever resemble those of dengue and other fevers caused by arthropod-borne viruses, confirmation of chikungunya fever should be based on: isolation of the virus, molecular methods, detection of IgM antibody, and demonstration of a rising titer of the IgG antibody [10]. In Bangladesh, only detection of IgM Ab is so far possible in Dhaka city. We confirmed our diagnosis by detecting IgM Ab against chikungunya virus. A specific antiviral agent or vaccine against chikungunya was not available till now. Treatment is supportive, involving rest, proper diet, movement and mild exercise. Combinations with mild pain relief medication, such as naproxen, ibuprofen, acetaminophen or paracetamol, may relieve the fever and aches. Re-evaluation and closer monitoring are advised in chronic ailments. Chikungunya virus infection provides immunity against the disease [11].

## Conclusion

Chikungunya fever is not uncommon in Bangladesh as evidenced by this short case series. Most of the cases remain undiagnosed or misdiagnosed due to lack of awareness and diagnostic facilities, the self-limiting nature of the disease and, most importantly, the prevalence of another arthropod-borne disease, dengue fever, in Bangladesh. Clinically, it can be distinguished from dengue fever but laboratory diagnosis is a prerequisite for confirmation.

## Consent

Written informed consents were obtained from our patients for publication of this case report. Copies of the written consents are available for review by the Editor-in-Chief of this journal.

## Abbreviations

Ab: Antibody; Anti CCP: Anti-cyclic citrullinated peptide; ICDDR,B: International Centre for Diarrhoeal Disease Research, Bangladesh; IEDCR: Institute of Epidemiology, Disease Control and Research; IgG: Immunoglobulin G; IgM: Immunoglobulin M; NSAIDs: Non-steroidal anti-inflammatory drugs; RF: Rheumatoid factor.

## Competing interests

The authors declare that they have no competing interests.

## Authors’ contributions

The first and corresponding author RH was involved in study design and in writing the manuscript. MMR was involved in the overall supervision of the study and writing the manuscript. MM was involved in data collection and manuscript writing. The other authors were involved in managing patients and data collection. All authors read and approved the final manuscript.
